# Return to work – estimated socioeconomic impact of spontaneous intracranial hypotension and effects of neurosurgical treatment

**DOI:** 10.3389/fneur.2026.1738826

**Published:** 2026-01-29

**Authors:** Mazin Omer, Katharina Wolf, Manou Overstijns, Amir El Rahal, Niklas Lützen, Horst Urbach, Charlotte Zander, Laura Krismer, Jan-Helge Klingler, Marc Hohenhaus, Mukesch Shah, Jürgen Beck, Florian Volz

**Affiliations:** 1 Department of Neurosurgery, Medical Center University of Freiburg, Freiburg im Breisgau, Germany; 2 Faculty of Medicine, University of Geneva, Geneva, Switzerland; 3 Department of Neuroradiology, Medical Center University of Freiburg, Freiburg im Breisgau, Germany; 4 Department of Diagnostic and Interventional Radiology, Medical Center University of Freiburg, Freiburg im Breisgau, Germany

**Keywords:** medical costs, socioeconomic effect, spinal CSF leak, spontaneous intracranial hypotension, working capacity

## Abstract

**Introduction:**

Spontaneous intracranial hypotension (SIH) due to a spinal cerebrospinal fluid (CSF) leak is a debilitating but curable condition, often affecting individuals of working age, making a considerably socioeconomic impact likely.

**Methods:**

This monocentric retrospective study in Germany analyzed work capacity in patients ≤65 years before and after surgical closure of a spinal CSF leak between April 2018 and September 2024. The economic burden was evaluated via direct hospital costs and indirect costs from productivity losses.

**Results:**

Two hundred and ten patients (median age 45.5 years, 62% female), all physically capable of working, were included. After symptom onset, 96% could not perform their professional work as before: 61% were completely unable, 18% reduced working time, and 17% adapted conditions. Three months postoperatively, 55% had completely returned to work, 19% were working part-time. At the last follow-up these numbers further improved to 65 and 17%, respectively, only 9% were still unable to work, 9% had retired. A shorter symptom duration was significantly associated with complete return to work. Median direct costs per patient for diagnosis and treatment was €11,407, indirect costs for 160 days (the median symptom duration before surgery) averaged €21,169. Extrapolated to the incidence rate, the additional annual economic burden in Germany is estimated at €85.75 million for 160 sick days, largely from preventable productivity losses.

**Conclusion:**

SIH significantly impairs working ability. Early treatment can restore work capacity and substantially reduces preventable productivity losses, strongly advocating timely intervention not only from a medical but also from an economic perspective.

## Introduction

Spontaneous intracranial hypotension (SIH) typically affects patients between 20 and 60 years of age (1–4) who were previously healthy with no relevant pre-existing conditions and therefore in the prime of their working lives (5, 6). Women are more frequently affected than men (1–5). Based on the estimated incidence rate of around 4/100,000 per year (1, 7), around 13,000 new cases can be expected each year in the United States, 3,200 in Germany, and 2,800 in the United Kingdom, respectively. SIH thus has an incidence rate comparable to aneurysmal subarachnoid hemorrhage (8, 9). In contrast to primary headache disorders such as migraine or cluster headache, SIH is an important secondary headache disorder with a clear and potentially treatable cause. The responsible spinal CSF leaks are traditionally classified into three types (5): ventral leaks with a dural tear anterior to the spinal cord, often caused by a discogenic microspur (10); lateral leaks with a dural tear in the vicinity of the spinal nerve root (11); and CSF-venous fistulas, a pathological direct connection between the thecal sac and a periradicular vein (12). Surgical closure of spinal CSF leaks has shown sustainable positive results, leading to significant improvements in patient reported outcomes for headache (13, 14), quality of life (15, 16), and mental health (15, 17). Next to these generic scores, an additional -however, not routinely implemented- practical measurement tool for the real-life impact of SIH is the assessment of work ability. In addition to its impact on an individual patient, SIH very likely exerts also a broader socioeconomic impact. This includes both direct costs spent on diagnosis and treatment of patients during hospitalization, as well as indirect costs resulting from productivity losses due to absence from work, loss of earnings, loss of tax revenue, and compensation payments. However, there have been no systematic studies on this to date. This study examines the impact of SIH on patients’ ability to work with the effect of surgical closure of the underlying CSF leak, and provides an estimate of the socioeconomic dimension of the disease.

## Methods

### Evaluation of work life impact and return-to-work timeline

This retrospective study from a tertiary referral center in Germany was approved by the local Ethics Committee (24-1296-S1-retro) and followed the STROBE guidelines (18). Patients aged ≤65 years who met the ICHD-3 criteria for SIH (19) and underwent surgery for a spinal CSF leak between April 2018 and September 2024 were evaluated for their ability to perform their professional work using an internally developed customized questionnaire (available in the Supplementary material). Four different points in time were assessed: before and after the onset of the SIH related symptoms, 3 months after surgery, and at the time of the survey (“last follow-up”). The working capacity before the start of symptoms was categorized in (1) working at full capacity, (2) voluntarily reduction of working hours, unrelated to physical limitations, (3) retired, and (4) unemployed. The status after the start of symptoms was categorized in (1) I work at full capacity, unchanged from previous workload, (2) I work at full capacity, but adapted (home office, laying down in the office, taking overtime etc.), (3) I reduced working capacity due to symptoms to 50–80%, or (4) to 20–50%, and (5) I am unable to work. If the questionnaire was not returned after two contact attempts, the patient was excluded from the study.

### Health economic analysis of direct and indirect costs

The total amount for diagnostics and subsequent surgical treatment reimbursed to the hospital by the respective health insurance was used as the reference for the direct cost calculations. Direct costs from previous external treatments (e.g., blood patch, surgical, or interventional procedures) were deliberately not included, as no reliable systematic data collection was possible. Indirect costs were estimated based on productivity losses due to work absence. These were calculated using the average gross monthly income in Germany of about €4,700, derived from publicly available data and published academic literature on income and sick leave policies (20–22). In accordance with the national sick-leave compensation scheme, the first 6 weeks of work absence were assumed to be covered at 100% by the employer (20), followed by income replacement at 70% by the statutory health insurance for a maximum of 78 weeks. After this period, patients typically transfer to disability pension or other forms of social welfare support (21). Additional losses due to uncollected taxes and social security contributions were included to estimate the total societal costs. The median symptom duration before surgery was used for the primary cost estimates and was modeled economically according to the German regulations, to reflect the actual legal framework. For comparison, cost estimates were exploratively calculated for a prolonged period of work incapacity of 360 days (1 year, labeled “delayed”) and for diagnosis and treatment after 90 days [labeled “early,” following previous studies (6, 23)] in order to illustrate both the economic burden of delayed diagnosis and the economic benefits of timely treatment.

### Statistical analysis

For statistical analyses, SPSS Statistics (IBM Corporation, version 29.0) was used. As all continuous variables showed a skewed distribution, they were presented as median with interquartile range (IQR). Changes in the ability to work were presented descriptively without specific statistical testing. Individual factors [sex, age, Body Mass Index (BMI), leak type, and symptom duration dichotomized in ≤90 days vs. >90 days] for returning to work at full capacity were investigated via a univariate logistic regression analysis and only variables reaching a significance level of *p* < 0.20 were included in the subsequent multivariable logistic regression. *p*-values <0.05 were considered statistically significant. All monetary values are reported in Euros. A sensitivity analysis was performed by varying key assumptions (average income, and sick-leave duration) within plausible ranges to assess the robustness of the cost estimates.

## Results

### Study population and demographics

**Figure 1:**
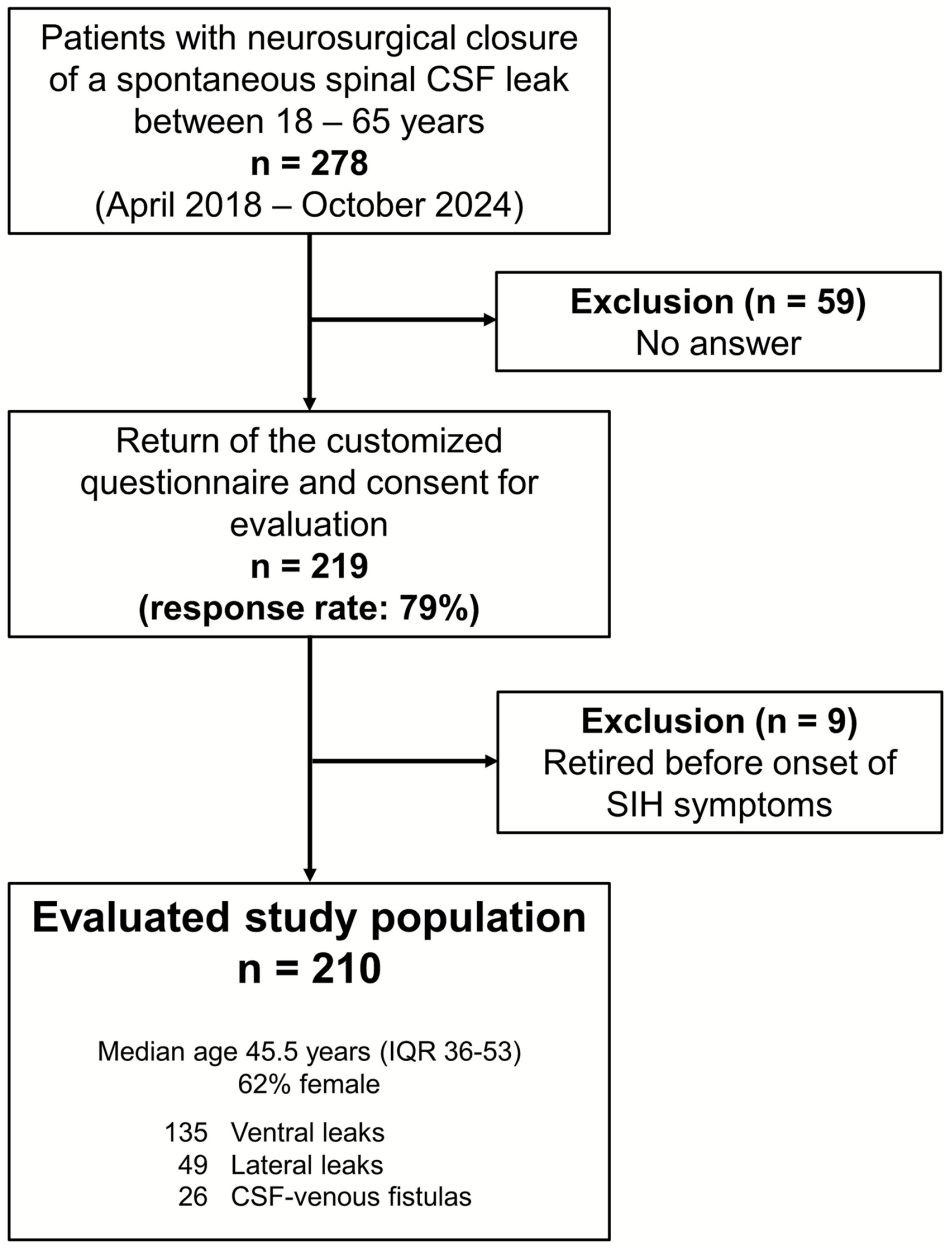
Flowchart displaying patients’ selection and demographics.

Between April 2018 and September 2024, a spontaneous spinal CSF leak was surgically sealed in 278 patients between the ages of 18 and 65. Of these, 219 (79%) returned the questionnaire. There were no significant demographic differences between responders and non-responders. After excluding nine patients who were already retired before the onset of the SIH related symptoms, 210 patients were included in the final analysis (Figure 1). The median age was 45.5 years (IQR 36–53, range 20–65 years), 130/210 (62%) were female. Median BMI was 23.8 kg/m^2^ (IQR 21.2–27.2, range 16.0–45.2 kg/m^2^). The majority of patients (174/210, 83%) lived in Germany and were insured under the German health insurance system. The remaining came from different, mainly European countries. Regarding the type of spinal CSF leak, 135/210 (64%) had a ventral leak, 49 (23%) had a lateral leak, and 26/210 (13%) had a CSF-venous fistula. The median duration of symptoms before the surgery was 160 days (IQR 83–493, range 7 days-24 years). The majority of patients (149/210, 71%) had a symptom duration longer than 90 days. Of the included 210 patients, 129 (61%) had received one or more epidural blood patches, typically externally, prior to transfer to our reference center. Twelve patients had already undergone one or more spinal surgeries with the intention of closing the leak externally prior to referral. Five patients had undergone unsuccessful radiological treatment including transvenous embolization and CT-guided targeted intervention.

### Surgical treatment and complications

All surgeries were performed in general anesthesia in prone position with minimally invasive tubular unilateral approach (24). For ventral leaks the transdural sandwich patching technique (25) was applied, for lateral leaks surgical resection of the arachnoid outpouching (11) with fibrin-patch augmentation and additional clipping (26) or suturing as appropriate was used, and for CSF-venous fistulas thermocoagulation of the draining veins with or without additional nerve root clipping (26) was applied. Fourteen of 210 patients (7%, 10 ventral leaks, and 4 lateral leaks) needed revision surgery: Two ventral leaks due to an epidural hematoseroma; two ventral leaks because of a dorsal dural closure insufficiency after the transdural approach; one ventral leak because of a superficial wound healing disorder; two ventral leaks with inconclusive intraoperative findings where the subsequently repeated imaging revealed the leak at an adjacent level; and seven patients (three ventral leaks and four lateral leaks) because of an insufficient sealing of the original leak, which was demonstrated via a repeated diagnostic workup within 10 days and 6 months. The routinely performed MRI of the brain and spine 3 months after the (last) surgery in addition to the clinical follow-up finally confirmed the successful sealing of the leak. Regarding permanent neurological deficits, except for an expectable unilateral dermatomal hypoesthesia after clipping of a thoracic nerve root, there was one case with a subtle gait ataxia (after transdural closure of a ventral leak in T2/3) and one case with a right sided hypoesthesia of the fingers 3–5 (after transdural closure of a ventral leak in C7/T1).

### Impact on working capacity

**Figure 2:**
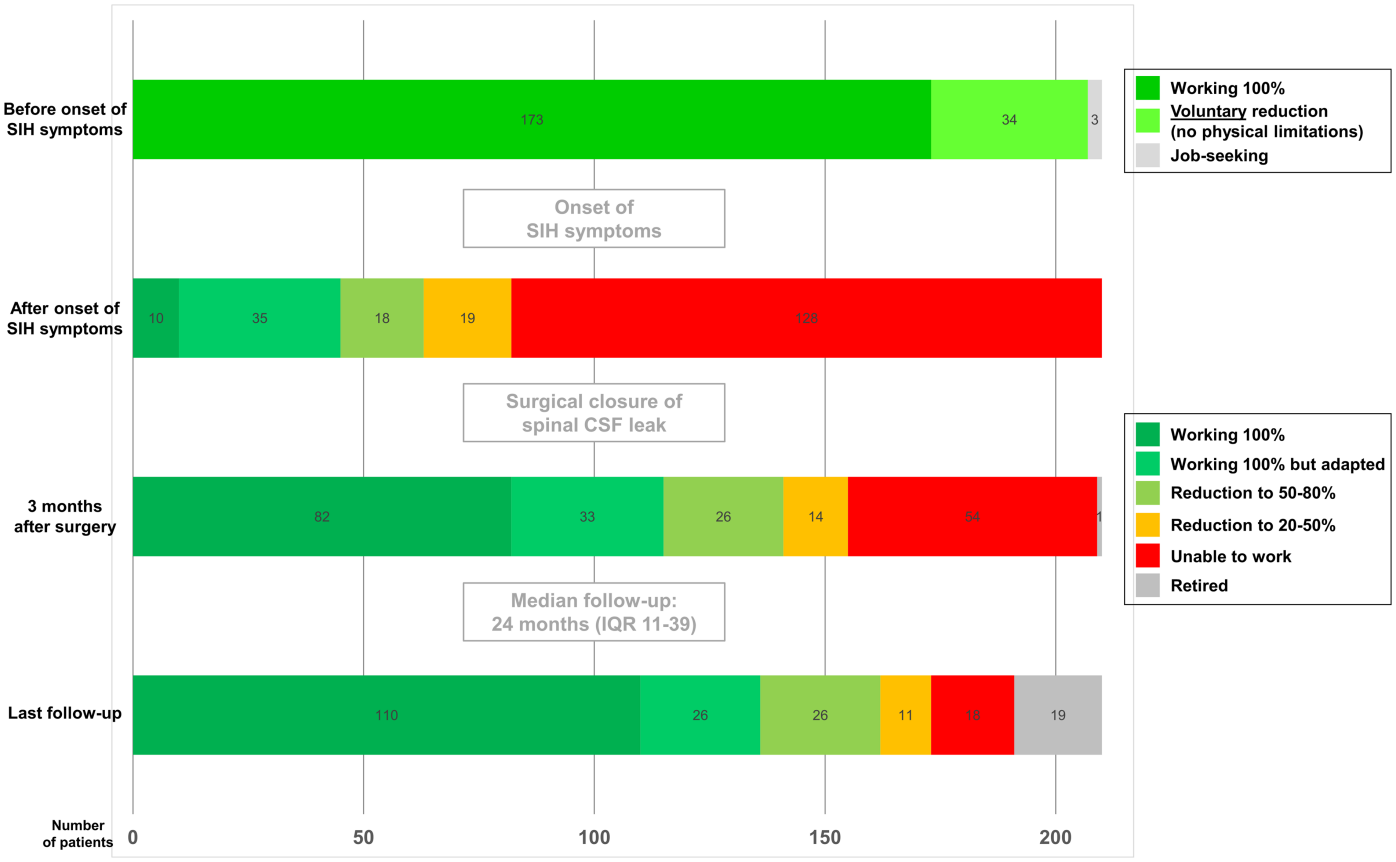
Bar graphs representing the ability to work in the study population of 210 patients at four different time points. Before symptom onset, 173 patients worked 100%, 34 reduced working hours voluntarily (not because of physical limitations), and 3 were unemployed. After symptom onset, 128 patients (61%) were completely unable to work, 37 (18%) had to reduce working hours. Only 45 patients (21%) were able to work full-time. Three months after surgery, there was a significant improvement: 115 patients (55%) have regained full working capacity, 40 patients at least reduced working capacity. Fifty-four patients (26%) were still completely unable to work. The last follow-up revealed further improvement: 136 patients (65%) now work at full capacity, working reduction was necessary for 37 patients. However, 18 patients (9%) were still completely unable to work.

Prior to the onset of SIH related symptoms, 173/210 (82%) patients worked full-time, 34/210 (16%) had voluntarily reduced their working hours, unrelated to physical limitations, and 3/210 (2%) were unemployed. After the onset of SIH related symptoms, most patients (128/210, 61%) were completely unable to work, 19/210 (9%) had to reduce their working hours to 20–50%, and 18/210 (9%) to 50–80%. Only 10/210 patients (4%) were able to work full-time, and another 35/210 patients (17%) worked at full capacity with adjustments of their working conditions (Figure 2). In summary, 96% of patients (200/210) were unable to perform their working capacity at the same level as before the onset of symptoms. Three months after surgery, there was a significant improvement. More than half the patients (115/210, 55%) were already able to perform their full working capacity, 82/210 (39%) completely comparable to before, and 33/210 (16%) with adjustments. Reduction of working capacity to 50–80% was necessary for 26/210 patients (12%), and to 20–50% for 14/210 patients (7%). However, 54/210 patients (26%) were still unable to work. The median follow-up time until the completion of the questionnaire (“last follow-up”) was 24 months (IQR 11–39 months, range 3–75 months). This last follow-up revealed further improvement: 136/210 patients (65%) could perform their full working capacity, 110/210 completely comparable to before, and 26/210 (12%) with adjustments. Reduction of working capacity to 50–80% was necessary for 26/210 (12%) patients, and to 20–50% for 11/210 (5%) patients. Finally, 18/210 (9%) patients were still completely unable to return to work at the last follow-up. After surgery, patients started returning to their professional work after a median of 6.5 weeks (IQR 4–12, range 1–96 weeks). The median duration until patients resumed their work at full capacity was 10 weeks (IQR 5–16, range 1–104 weeks). Given the heterogeneous follow-up duration (range 3–75 months), a sensitivity analysis stratified by follow-up ≤12 months versus >12 months was performed. Patients with longer follow-up showed higher rates of full work capacity and lower rates of inability to work, indicating continued recovery over time. Importantly, the overall work-capacity distribution at last follow-up remained consistent with the main cohort analysis, supporting the robustness of the reported outcomes (Table 1). Nineteen patients retired during the follow-up period. In the univariate logistic regression only sex and symptom duration (dichotomized in ≤90 days vs. >90 days) were significantly associated with the return to work. Age, BMI, and the type of CSF leak showed no significant association. Multivariable logistic regression analysis including sex and symptom duration revealed that male patients had three times higher chance to return to work (OR = 3.048; 95% CI [1.653, 5.620], *p* = 0.0003) than female patients. Patients with a symptom duration ≤90 days had a 2.5-fold chance to return to work (OR = 2.494; 95% CI [1.282, 4.851], *p* = 0.007) than patients with longer symptoms.

**Table 1:** Work capacity at last follow-up stratified by follow-up duration (≤12 months vs. >12 months).

Follow-up duration	Full capacity	Reduced capacity	Unable to work	Retired/other
≤12 months	59.0%	23.0%	14.8%	3.2%
>12 months	67.1%	15.4%	4.7%	12.8%

### Socioeconomic burden of SIH

**Figure 3:**
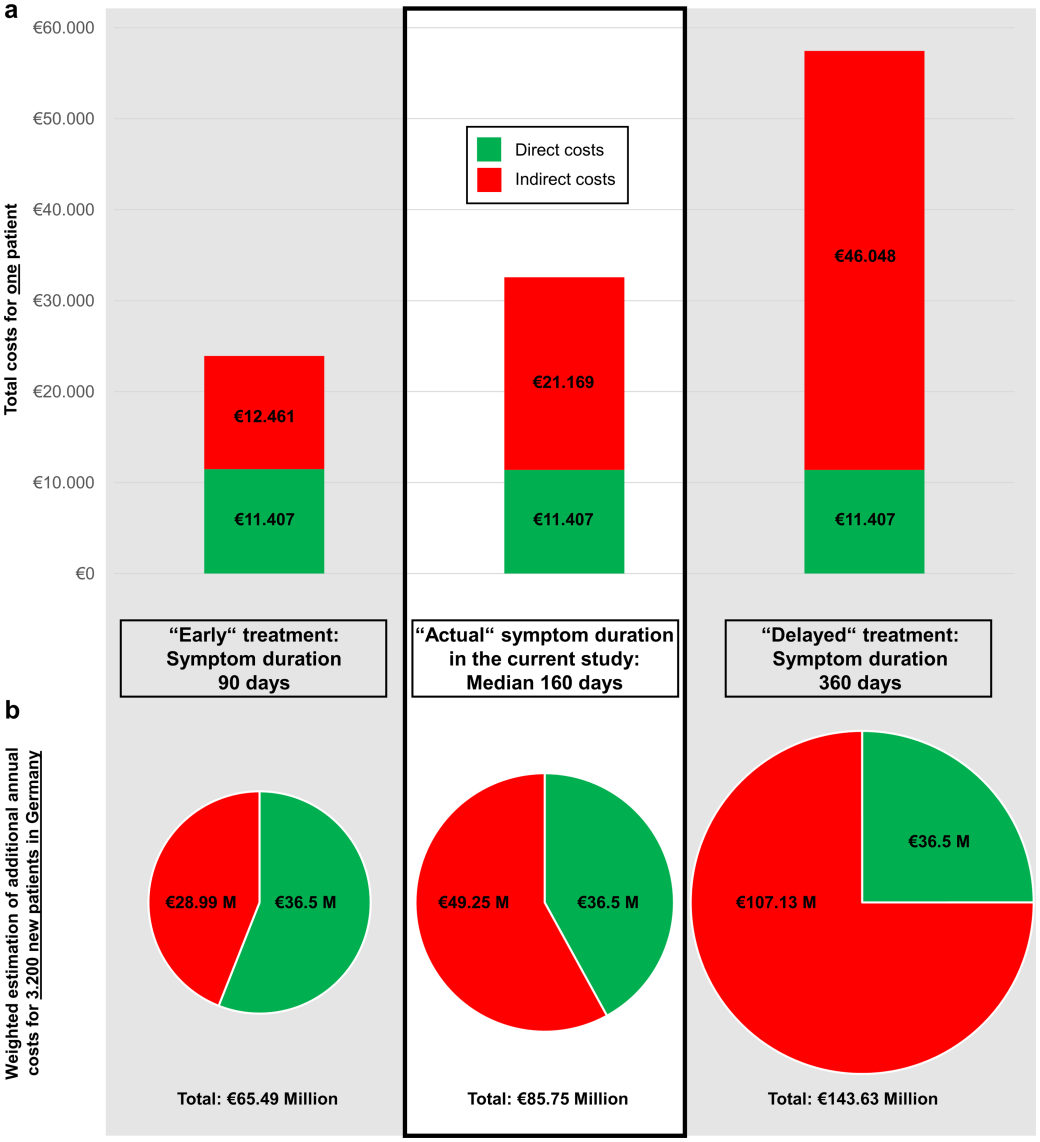
Estimated total costs for three different time periods before diagnosis and treatment of spontaneous intracranial hypotension (SIH): “Early” treatment is defined as diagnosis and treatment within 90 days after symptom onset. “Actual” treatment of 160 days is the median symptom duration before surgical treatment in the study population (thus representing the estimated costs of the actual cohort). “Delayed” treatment was deliberately defined as 360 days to illustrate the estimated costs in 1 year. **(a)** Bar graph for the total costs per patient. **(b)** Pie charts for the estimated new annual economic burden of SIH in Germany. The area of each circle is representative for the actual amounts. Based on an estimated annual incidence of ~3,200 new cases every year in Germany, our results correspond to a weighted national economic burden of €85.75 million, with €49.52 million from indirect costs. With increasing diagnostic and treatment delay, direct costs remained constant (€11,407), while indirect costs rose sharply—from €21,169 per patient at 160 days to €46,048 at 360 days—leading to a corresponding increase in the national economic burden from €85.75 million to €143.63 million. Earlier diagnosis at, e.g., 90 days reduced indirect costs to €12,461 per patient and the national burden to €65.49 million.

The economic analysis was structured into two periods: a pre-referral phase, representing the time before diagnosis and initiation of treatment, during which most indirect costs arise, and a hospital phase, encompassing diagnostic work-up and definitive surgical closure of the leak. During the median pre-referral period of 160 days, indirect costs averaged €21,169 per patient (sick leave and compensation payments). The hospital phase, including inpatient stay, diagnostic imaging, and surgery, accounted for a median of €11,407 per patient (IQR 10,801–12,373; range €8,880–21,958). The total per-patient cost was €32,576, with 65% attributable to indirect costs in the pre-referral period (Table 2; Figure 3a,b).

**Table 2:** Direct costs (derived from the controlling department of medical center), and estimated indirect costs of spontaneous intracranial hypotension (SIH) per patient, based on the median symptom duration of 160 days before treatment in the study population.

Category	Details	Cost
Direct costs		
Hospital treatment (median)	Covers inpatient stay, imaging, surgery	€11,407 (IQR 10,801–12,373)
Total direct costs (median)		€11,407
Indirect costs (average)		
Employer sick pay (first 6 weeks)	Paid by employer (100% salary for 42 days)	€6,490 ^[20]^
Statutory insurance sick benefit	Paid by statutory health insurance (70% salary for 118 days after the first 42 days)	€12,764 ^[21]^
Loss of taxes and contributions	Uncollected tax and social security revenue	€1,915
Total indirect cost		€21,169
Total costs (per patient)	Based on the median symptom duration of 160 days	€32,576

**Figure 4:**
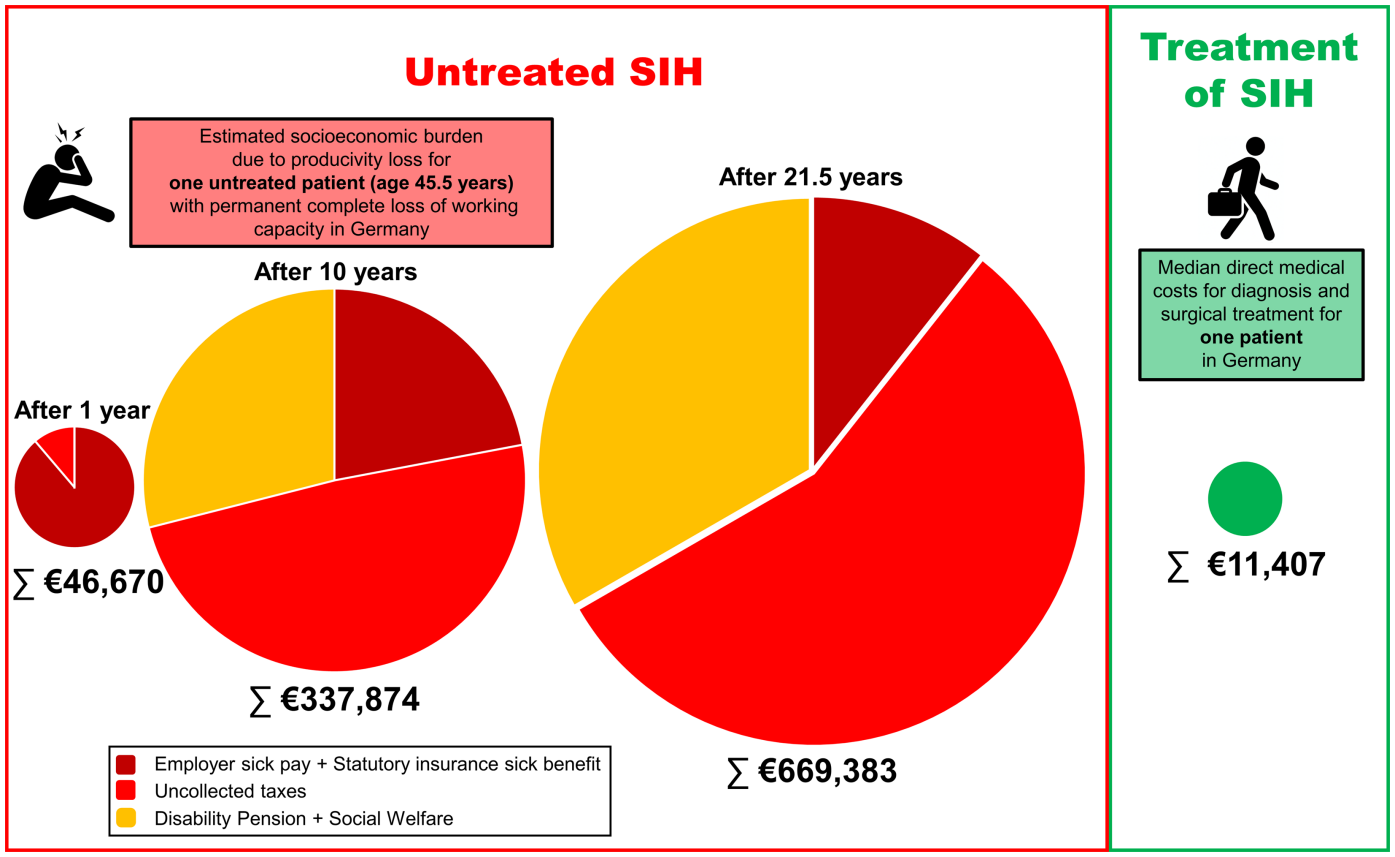
Pie chart of the estimated costs for one individual patient of 45.5 years (median age of our cohort) based on the legal framework in Germany. The area of each circle is representative for the actual amounts. Assuming permanent total incapacity to work until retirement after 21.5 years, the indirect costs due to productivity losses (including compensatory payments and loss of taxes) rise continuously. In comparison, the direct (medical) costs for diagnosis and surgical treatment are significantly lower.

**Figure 5:**
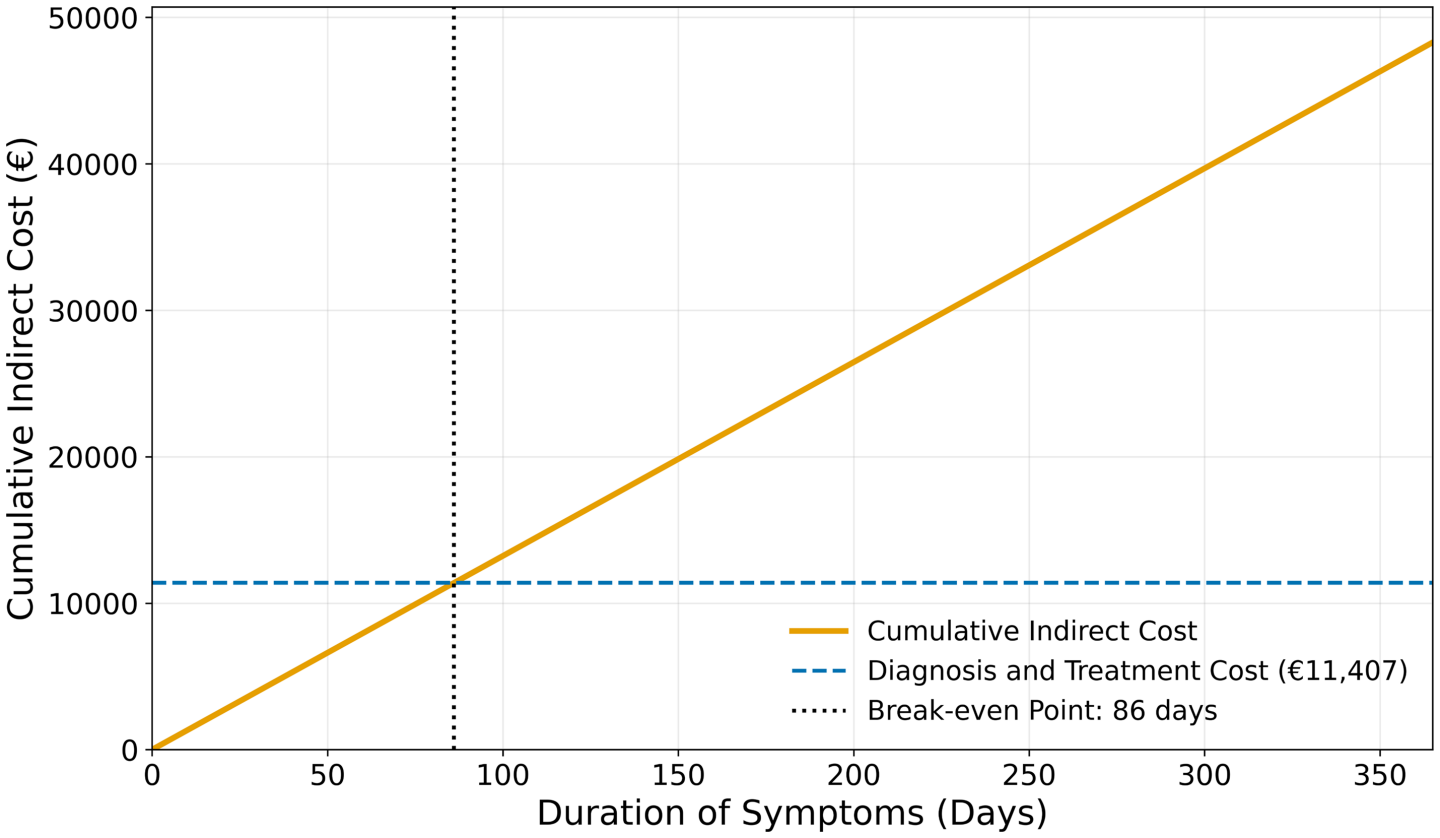
Adjusted break-even point for cost-effective intervention in spontaneous intracranial hypotension based on the German social insurance framework.

The economic model in Figure 4 illustrates the long-term burden of SIH for a single patient with the cohort’s median age of 45.5 years and persistent incapacity to work. Assuming continued work-incapacity over 10 years, indirect costs accumulated to €337,874, and to €669,383 until the current statutory retirement age in Germany at 67 years (21.5 years from the median age). Figure 5 shows that indirect costs increased almost linearly with the duration of undiagnosed and untreated SIH, making early intervention more cost-effective from a societal perspective. Indirect costs exceeded the average direct medical costs of €11,407 after approximately 86 days, marking a purely economic break-even point. Sensitivity analysis varying the indirect costs by ±20% demonstrated that prolonged illness duration was the dominant driver of total costs (Table 3). Across all scenarios, early definitive treatment within 90 days resulted in substantially lower total costs (€21,376–€26,360) compared to the cohort median duration (160 days; €28,342–€36,809) and prolonged cases (360 days; €48,245–€66,664), underscoring the robust economic benefit of timely intervention.

**Table 3:** Sensitivity analysis of total per-patient costs by illness duration and indirect cost variation.

Scenario	Duration (days)	Indirect costs (€)	Total costs incl. Direct costs (€)
–20% (indirect costs)	90 days	€9,968.80	€21,375.80
	160 days	€16,935.20	€28,342.20
	360 days	€36,838.40	€48,245.40
Base (component model)	90 days	€12,461.00	€23,868.00
	160 days	€21,169.00	€32,576.00
	360 days	€46,048.00	€57,455.00
+20% (indirect costs)	90 days	€14,953.20	€26,360.20
	160 days	€25,402.80	€36,809.80
	360 days	€55,257.60	€66,664.60

## Discussion

This study demonstrates for the first time the high socioeconomic relevance of SIH. After a remarkably high response rate of 79% the study includes 210 patients, resulting in a large and representative study population. After the onset of SIH symptoms almost all patients (96%) were not able to perform their professional work as before. Surgical closure of the underlying CSF leak leads to a significant improvement, with more than 80% of patients returning to work completely or at least partially. Compared to the high indirect costs, particularly due to compensation payments and productivity losses the direct medical costs are significantly lower. Our results emphasize the importance of timely diagnosis and treatment, not only from the individual patient’s perspective but also from the socioeconomic point of view.

Although the overall results are very positive, still 9% of patients remained permanently unable to work exclusively due to SIH. These results confirm those of the previous smaller cohort with only 34 patients (26) and resemble those of patient-reported outcome studies, in which one quarter of patients reported relevant headaches (14) or depressive symptoms (17) even after successful leak closure. Also comparable to these studies is the fact that recovery takes time after successful leak closure. While there is already a significant improvement in working ability after 3 months, the results continue to improve significantly until the last follow-up. This fact should be communicated early on to the affected patients as well as to their social and work environment. Previous studies have shown that a shorter symptom duration ≤90 days prior to surgery is associated with better outcomes (6, 23). The present study confirms these results, this time with a finding that is highly relevant not only for patients themselves but also for society: When treated within 90 days, the odds of returning to work were more than two times higher. Especially since the time until definitive treatment remains the only modifiable factor, this correlation cannot be emphasized enough and should be included in future treatment guidelines.

To accurately determine the total costs of a disease for society, it is necessary to know its prevalence, that is, the exact number of people affected at a given point in time. However, these figures are not available for SIH, but a high number of undiagnosed cases must be suspected. At present, the estimated annual incidence rate is 4/100,000 (1, 7), resulting in approximately 3,200 new cases per year in Germany. Based on our numbers this leads to additional total costs of approximately 104 million each year in Germany. Considering the high number of undiagnosed cases, the actual costs of untreated SIH are certainly much higher.

When interpreting the presented direct and indirect costs, several fundamental aspects must be taken into account, which also illustrate certain limitations in the methodological approach we used: The direct costs represent a “best-case scenario” due to the high level of experience at the treatment center, which results in exceptionally low costs, and because previous treatment costs were not considered. A potentially higher revision rate in less experienced centers might possibly lead to higher direct costs. On the other hand, the estimated indirect costs represent a “worst-case scenario,” assuming the patient remains permanently unable to work. This contrasts with the observation that clinical symptoms might improve slightly spontaneously or after epidural blood patching, and that a return to work is sometimes possible. Considering these possibilities, that spontaneous recovery with or without epidural blood patching, or a partial return to work might occur, the presented cost estimates are potentially too high. Thus, further specially designed and prospective socioeconomic analyses are clearly needed. Finally, the estimated costs only apply within the German healthcare and social system. In other countries, the access to adequate and immediate treatment might be limited, and costs could differ significantly. Regardless of this, we believe that the presented results give a clear impression of the significant socioeconomic impact of SIH and the potential of surgical treatment.

Furthermore, this study demonstrates that SIH—despite being a treatable and often reversible condition—produces a cost profile comparable to other chronic high-burden disorders including chronic pain conditions, with indirect costs, accounting for most of the total burden. Published cost-of-illness studies report mean annual per-patient costs of approximately €1,322 for chronic low back pain (27, 28), €12,000 for cluster headache (29), €14,446 for trigeminal neuralgia (30), and €6,700 for migraine (31), reflecting the chronic and recurrent nature of these conditions. This finding is particularly noteworthy because, unlike those chronic conditions where indirect costs may currently be largely unavoidable, the economic losses in SIH are potentially preventable through timely diagnosis and surgical treatment. Large-scale European analyses, such as the COIN-EU project, have shown that brain disorders account for an outsized share of healthcare and societal costs, with indirect costs—mainly due to lost productivity—often surpassing direct medical expenses (32). Similar findings from Danish registry data confirm that the economic drain from neurological disorders is increasingly driven by workforce withdrawal rather than hospital expenditures (33), reflecting the importance of taking this aspect into account when tackling these conditions. However, in contrast to other conditions where indirect costs are harder to avoid due to residual untreatable symptoms, most indirect losses in SIH stem from preventable treatment delays. This positions SIH as a rare example of a high-burden neurological condition where targeted health system changes – namely faster diagnosis and referral to specialized centers – could yield immediate and measurable economic gains, alongside restoring functional independence for a working-age population. The productivity-related losses are shouldered by multiple stakeholders: employers (who cover wages during the initial 6-week sick leave), social insurers (who pay statutory sick benefits for extended leave), and the public sector (through uncollected tax and social security revenues). In practical terms, SIH represents a significant loss of economic output, roughly equivalent to removing thousands of working days from the labor market each year. Faster diagnosis and treatment would directly cut these indirect costs, underscoring how timely intervention can yield not just clinical benefits but also economic savings. Based on the findings from our study, we recommend that health systems prioritize early recognition and prompt referral of suspected SIH cases to specialized centers as early as possible, to maximize both clinical recovery and economic efficiency. This could happen by establishing standardized diagnostic pathways, increasing awareness among primary care and emergency physicians, and by ensuring rapid access to advanced imaging and surgical expertise. To achieve that, the establishment of specialized and dedicated centers with a high case load is essential.

## Limitations

Beside the inherent limitations of a retrospective, monocentric study there are additional limitations: First, response and recall bias are theoretically possible. However, a response rate of 79% and a clearly defined objective (“able to work or not”) make both rather unlikely. Second, due to the substantial experience in our center, there is an obvious selection bias and the results cannot be readily generalized. Additionally, the study focuses on surgically treated patients and therefore does provide a general assessment of all treatment options, including epidural blood patching. The reported direct medical costs might be regarded as a “best case scenario” and higher costs (e.g., due to more frequent readmissions and revision surgeries) might be expected at less experienced centers, which further emphasizes the high socioeconomic relevance. Third, the deliberate lack of consideration of external costs from previous treatments and from known sequelae of SIH like cerebral siderosis or subdural hematomas leads to a systematic underestimation of direct medical costs. This however, again rather highlights the high socioeconomic aspect of SIH. Fourth, our economic extrapolation assumes that all new SIH patients are of working age and become fully unable to work due to the disease. Last, as the calculated costs apply only to the German healthcare system, the actual amounts may vary accordingly in other countries. Indirect costs were restricted to the pre-treatment period; therefore, persistent productivity losses during the early postoperative phase fall outside the scope of the current study and may not be fully captured. Regardless of that, the fundamental impact of SIH on the ability to work remains severe.

## Conclusion

SIH regularly causes a loss of working ability in previously healthy and full-time working patients. Surgical closure of the responsible spinal CSF leak can restore working ability in the majority of cases. The high economic burden is driven largely by preventable indirect costs coming from productivity losses during prolonged sick intervals. In experienced centers the direct medical costs for diagnosis and treatment are relatively low and clearly beneficial from an economic perspective. Prompt successful treatment not only improves the clinical outcome but also reduces the indirect medical costs. Our findings highlight that accelerating access to definitive treatment not only restores patients’ work life but also yields substantial economic benefits for society. The assessment of working capacity before and after treatment should be included in patients’ standard evaluation and in comparisons between different treatment modalities.

## Data Availability

The raw data supporting the conclusions of this article will be made available by the authors, without undue reservation.
