# Supplementary material for: Return to work – estimated socioeconomic impact of spontaneous intracranial hypotension and effects of neurosurgical treatment
Source: Front Neurol. 2026 Jan 29;17:1738826. doi: 10.3389/fneur.2026.1738826 (PMC12894015; doi:10.3389/fneur.2026.1738826)
Supplement: Supplementary file 1 [file Supplementary_file_1.pdf]

Date of birth: (day/month/year): _______/______/_______

If you remember: **Start of CSF loss symptoms**: (day/month/year): _____/____/_____

Before the start of those symptoms, I was

□ working/employed, at ________ % (Profession: ______________________________________)
□ retired
□ unemployed

After the start of those symptoms

□ I could work at full capacity, unchanged from previous workload
□ I worked at full capacity, but adapted (homeoffice, laying down in the office, taking overtime etc.)
□ I reduced working capacity due to symptoms to 50-80%
□ I reduced working capacity due to symptoms to 20-50%
□ I was unable to work / on sick leave

After surgical treatment:

When did I **start** working again after the surgery: ____________ weeks/months/years

When after the surgery I was able to **work 100% again**: _____________ weeks/months/years

3 after the surgery:

□ I could work at full capacity, unchanged from previous workload
□ I worked at full capacity, but adapted (homeoffice, laying down in the office, taking overtime etc.)
□ I reduced working capacity due to symptoms to 50-80%
□ I reduced working capacity due to symptoms to 20-50%
□ I was unable to work / on sick leave

Currently:

□ I work at full capacity, unchanged from previous workload
□ I work at full capacity, but adapted (homeoffice, laying down in the office, taking overtime etc.)
□ I reduced working capacity to 50-80%
□ I reduced working capacity to 20-50%
□ I am unable to work / on sick leave
□ retired
□ unemployed

If **not** fully capable of working:

□ because of symptoms attributable to CSF problems
 □ low pressure (hypotension) □ high pressure (“*rebound hypertension*”)
□ because of local back pain around the surgery site
□ because of other complaints. Please specify: ________________________________

Thank you very much for your participation.

Prof. Dr. Jürgen Beck **Dr. Florian Volz** **Dr. Amir El Rahal**

By returning this letter, I agree to the anonymized collection and evaluation of my data. It is not possible to draw any conclusions about my person or my case. You can find detailed information on the handling of your data at https://www.uniklinik-freiburg.de/footernavigation/datenschutz.html
